# Long-Term Atmospheric Visibility Trends and Their Relations to Socioeconomic Factors in Xiamen City, China

**DOI:** 10.3390/ijerph15102239

**Published:** 2018-10-12

**Authors:** Weicong Fu, Qunyue Liu, Cecil Konijnendijk van den Bosch, Ziru Chen, Zhipeng Zhu, Jinda Qi, Mo Wang, Emily Dang, Jianwen Dong

**Affiliations:** 1 College of Landscape Architecture, Fujian Agriculture and Forestry University, Fuzhou 350002, China; weicongfufj@163.com (W.F.); fafulqy@gmail.com (Q.L.); fjchenziru@126.com (Z.C.); zhuzhipeng512@126.com (Z.Z.); 2 Urban Forestry Research in Action, Department of Forest Resources Management, The University of British Columbia, Vancouver, BC V6T 1Z4, Canada; cecil.konijnendijk@ubc.ca; 3 Collaborative for Advanced Landscape Planning, Faculty of Forestry, The University of British Columbia, Vancouver, BC V6T 1Z4, Canada; 4 Faculty of Forestry, The University of British Columbia, Vancouver, BC V6T 1Z4, Canada; emily.dang@hotmail.com; 5 Faculty of built environment, University of New South Wales, Sydney 2052, Australia; jinda.qi@student.unsw.edu.au; 6 College of Architecture & Urban Planning, Guangzhou University, Guangzhou 510006, China; landwangmo@outlook.com

**Keywords:** residents’ activities, industrial activities, structural equation model, air quality

## Abstract

Atmospheric visibility (AV), one of the most concerning environmental issues, has shown a continuous decline in China’s urban areas, especially in Southeastern China. Existing studies have shown that AV is affected by air pollutants and climate change, which are always caused by human activities that are linked to socioeconomic factors, such as urban size, residents’ activities, industrial activities, and urban greening. However, the contribution of socioeconomic factors to AV is still not well understood, especially from a long-term perspective, which sometimes leads to ineffective policies. In this study, we used the structural equation model (SEM) in order to quantify the contribution of socioeconomic factors on AV change in Xiamen City, China, between 1987–2016. The results showed that the annual average AV of Xiamen between 1987–2016 was 12.00 km, with a change rate of −0.315 km/year. Urban size, industrial activities, and residents’ activities were found to have a negative impact on AV, while the impact of urban greening on the AV was modest. Among all of the indicators, the number of resident’s vehicles, total retail sales of consumer goods, and household electricity consumption were found to have the highest negative direct impact on the AV. The resident population, urban built-up area, and secondary industry gross domestic product (GDP) were the most important indirect impact factors. Based on our results, we evaluated the existing environmental regulations and policies of Xiamen City.

## 1. Introduction

According to the United States Environmental Protection Agency (USEPA), the atmospheric visibility (AV) of the non-polluted atmosphere varies from 145 km to 225 km in different areas [1]. AV is determined mostly by the light extinction of air pollutants, and the influence factors of AV are air pollutants and the meteorological condition [2,3,4,5,6]. Studies have also shown that the variation of AV is linked to public health [3,4,5,6]. Huang and Tan et al. (2009) found that poor AV is associated with total and cardiovascular mortality in Shanghai, China [7]. Wang et al. (2013) also found that a poor AV significantly affects public emotion and reduces the sensory experience of visitors [8]. For many decades, urban areas have been facing an AV decline due to rapid economic development, which is especially true for China [9,10,11]. Studies in China have shown a straightforward picture of the characteristics of AV. Chen et al. (2016) analyzed the long-term data of AV in Chengdu and Chongqing, and found that, since the 1970s, the AV has been lower [12]. Deng et al. (2012) found that in the rural and urban areas of Southeast China, the AV decreased at a trend of 2.0 km/decade between 1973–2010 [13]. Huang et al. (2016) revealed that the annual average AV of Hefei City was generally lower than 7 km, and it showed a worsening trend from 2001 to 2006 [14].

Therefore, questions about what impacts AV as well as what these mechanisms are, have evoked great attention from the public, scientists, and policy makers [8,9,10,11,12,13,14,15,16,17,18,19,20]. Kuo et al. (2013) revealed that the AV was low in urban areas as a result of the lower wind speeds in urban areas, which are influenced by urban area expansion and tall high-density buildings. They also revealed that great amounts of aerosols (e.g., oxides of nitrogen (NOx) and volatile organic compounds (VOCs)), usually emitted from vehicles and residents’ cooking, lead to an AV decline in urban areas [18,19]. Cao et al. (2012) and Chen et al. (2012) inferred that a poor AV had a strong correlation with the industries in Xi’an and the Sichuan Basin [20,21]. Vos et al. (2013), Nowak et al. (2014), and Selmi et al. (2016) reported that green spaces can mitigate air pollutants and can buffer declining AV trends [22,23,24].

As mentioned above, many studies have reported that the change of AV in urban areas is significantly linked to socioeconomic factors, such as urban size (US), residents’ activities (RA), industrial activities (IA), and urban greening (UG) [7,8,16,17,25]. Nevertheless, to date, very few studies have analyzed the contribution of socioeconomic factors on AV directly. Furthermore, no studies have analyzed the effects of socioeconomic factors on AV in the long term (e.g., more than 20 years). In terms of the existing studies, the sort-term data from a specific region or an entire country, such as China, have been used to examine the relationships between the AV and socioeconomic factors [26]. However, in different cities and different study areas, because of the difference in the socioeconomic composition, terrain, and climate characteristics, the socioeconomic factors that affect the characteristics of AV are not the same. Moreover, so far, there has been a singular focus on the negative factors, while the positive factors (e.g., greenspace) that can reduce AV decline have generally been ignored [26]. The lack of a comprehensive understanding of the socioeconomic causes of urban AV decline prevents the government and the public from conducting environmental improvement activities. To make policies and environment improvement activities more effective, there needs to be an understanding of how socioeconomic factors impact AV in the long-term.

This study focuses on Xiamen City, a sub-tropical monsoon city in the coast area of Southeastern China, where the annual temperature, relative humidity, rainfall, and wind speed are 21.4 °C, 82%, 2168.2 mm, and 2.7 m/s, respectively. Xiamen is one of the most developed and densely populated cities in Southern China. As a world-renowned city, Xiamen has hosted many international conferences, such as the G20 Ministerial Meeting in 2016, and the BRICS (Brazil, Russia, India, China, and South Africa) Conference in 2017. Xiamen has been undergoing accelerated economic growth, urbanization, industrial expansion, and population growth. It is expected to experience further dramatic urbanization for the foreseeable future. The recent fast economy growth and urbanization have led to increasing air pollution and declining AV. In this study, the socioeconomic data and AV data during the period of 1987–2016 were employed so as to investigate the long-term relationship between the AV trends and socioeconomic factors. We tried to answer the following three questions: (1) How do different socioeconomic factors affect the AV? (2) How can these effects be quantified? (3) What recommendations can we give to policy makers and the public based on our research?

## 2. Materials and Methods 

### 2.1. Data Collection and Analysis

**Figure 1:**
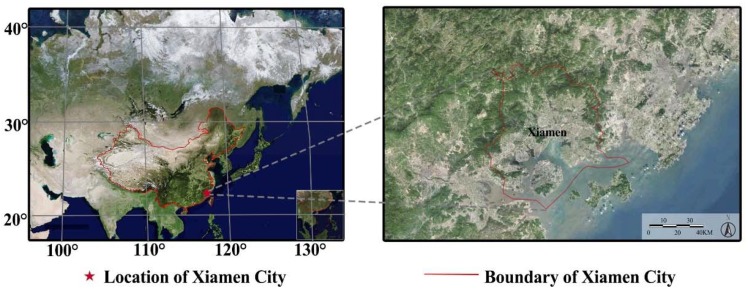
Location of Xiamen city, China (source: Google Maps).

The geographical location of Xiamen is depicted in Figure 1. Well-trained operators have measured AV using easily identifiable objects (e.g., tall buildings and mountain ridges) at predetermined distances. The AV and meteorological hourly data were obtained through the official release from the National Oceanic and Atmospheric Administration of America (NOAA; https://www.climate.gov). NOAA is respected as an authority that develops data sets on meteorological information from all over the world. The monitoring sites in Xiamen are managed by the China Meteorological Administration. Two statistical approaches were utilized in this study in order to investigate the tendency of AV during the study period, namely: (1) the calculation of trends of AV, and (2) the statistics of the trends of rates with a “good” AV (≥20 km) and “bad” AV (<10 km). The latter was used, as an AV of 10 km is an important indicator of haze pollution in China and an AV of 20 km is an important indicator of high AV, mentioned by aforementioned studies [27,28]. The chosen investigation methods are also elaborated in previous studies [27,28,29,30].

The socioeconomic variables were compiled from the Xiamen Statistics Yearbooks from 1986–2017, the China Urban Construction Statistics Yearbooks from 1986–2017 (National Bureau of Statistics, 1986–2017), and municipal official websites. The trends of all of the socioeconomic data were also analyzed in this study.

### 2.2. Model Development and Analysis

A structural equation model (SEM) was used to analyze the relationship between the AV and socioeconomic factors in this study. The use of SEM was motivated by its usefulness for investigating complex networks of relationships, and of its conformation as a means of representing theoretical concepts using latent variables [26,31]. It was used to estimate the multiple latent variables at once, while accommodating measurement errors [26,31]. These features allowed us to assess the influences of socioeconomic factors on AV and to compare their relative contributions.

Previous studies revealed that anthropogenic emissions were the main sources of AV decline, including industrial production (e.g., secondary industry gross domestic product (GDP), industrial waste gas, industrial dust emissions, Sulphur dioxide emissions, and industrial electricity consumption), residents’ emissions (e.g., emissions of vehicles, residents’ electricity consumption, and the total retail sales of consumer goods), and city size (including urban built-up areas and resident populations) [8,32,33,34,35,36]. According to previous studies, urban size, residents’ activities, industrial activities, and urban greening (UG is an important part of urban areas, previous studies that have focused on air pollutants take UG as an important socioeconomic factor [22,23,24] were the four socioeconomic factors that impacted the variation of AV (Table 1). In our model, these four socioeconomic factors were set as the explanatory variables, and the AV was set as the dependent variable. We selected indicators for each socioeconomic variable by the following two criteria: (1) The selected indicator must affect AV directly or indirectly (i.e., the influence of these indicators on AV has been shown by existing studies). (2) The data for these indicators must be available for Xiamen City. According to the requirements mentioned above, thirteen indicators were selected after screening through the literature (Table 1).

**Figure 2:**
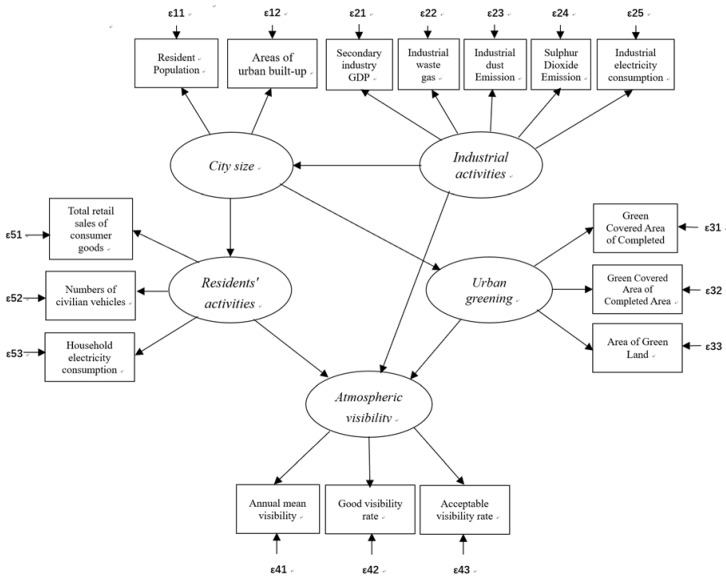
The initial model used for the study. The variables in rectangles are the observed factors; the variables in the ellipses are the latent variables; **ε** represents the errors of the observed factors.

In our model, the AV was measured with the following three indicators (Figure 2): the annual mean value, the rate of good AV (i.e., annual percentage of the hours with the hourly average AV higher than 20 km), and the rate of acceptable AV (i.e., annual percentage of the hours with the hourly average AV higher than 10 km, as an AV of 10 km is an important indicator of haze pollution in China) (China Meteorological Administration, 2010). Most studies only used the annual average value as an indicator of AV [25,34,38]. This cannot reveal the real characteristics of AV. For example, the annual mean AV in Guangzhou, Nanning, and Changchun in 2015 were 8.73, 8.89, and 8.99, respectively (i.e., almost the same). Nevertheless, the rates of good AV in Guangzhou, Nanning, and Changchun were 16.16%, 20.45%, and 30.73%, respectively. Epidemiological studies have shown that a longer exposure to haze (AV < 10 km, mainly caused by high particular meter concentrations, which is reveal by industries and vehicles in China) was more harmful to human health than short exposure [3,4,5,6]. The inclusion of the rate of haze time also helped to describe the AV more accurately. For example, the annual mean AV in 2009, 2010, and 2011 in Beijing was 11.51, 11.28, and 11.24 km. However, after three years, the rates of haze times had changed (i.e., 11.01, 13.49, and 14.26%, respectively). This is part of the reason that the government announced that air pollution has been lowered (Beijing Municipal Environmental Protection Bureau, 2016), although the public felt that it had become worse.

We took three steps in developing our model. (1) We constructed a preliminary SEM model. In this model, urban size, residents’ activities, industrial activities, and urban greening were the four socioeconomic explanatory factors, and AV was the dependent variable (Figure 2). (2) SPSS (version 23.0, IBM, Armonk, NY, United States), AMOS (version 24.0, IBM, Armonk, NY, United States), and R (version 3.4.4, R Foundation for Statistical Computing, Vienna, Austria) were used to undertake the validation analysis (i.e., correlation analysis, reliable test, validity analysis, and Cronbach’s coefficient) and to make the SEM fit [26]. (3) We modified the SEM using the method that existing studies had previously used [26].

## 3. Results

### 3.1. Trends and Characteristics of Atmospheric Visibility and Socioeconomic Factors

#### 3.1.1. Atmospheric Visibility

Table 2 summarizes the mean AV during the periods of 1987–1991, 1992–1996, 1997–2001, 2002–2006, 2007–2011, 2012–2016, and 1987–2018, as well as the overall trend of the AV in Xiamen. As shown in Table 2, the mean value of AV in Xiamen was 12.00 km, with a declining rate of −0.315 km/year during 1987–2016. The mean values of the AV during 1987–1991, 1992–1996, 1997–2001, 2002–2006, 2007–2011, and 2012–2016 were 17.14, 13.92, 11.52, 10.54, 10.55, and 8.33 km, respectively, with changing rates of −0.15, −0.98, 0.02, −0.20, 0.001, −0.61, and −0.315, respectively. This indicated that the AV in Xiamen was poor and was getting worse.

**Figure 3:**
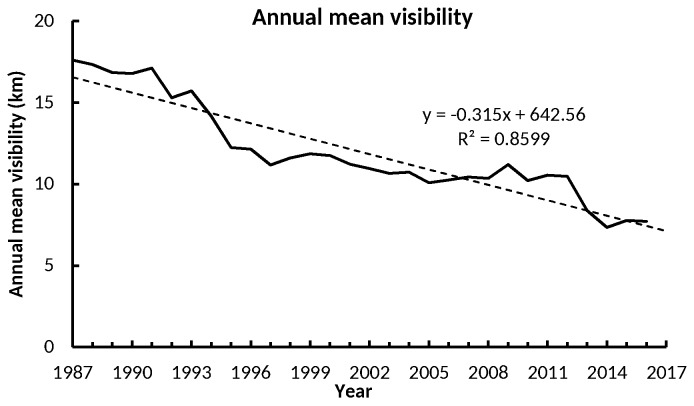
Variation of annual mean visibility (km) of Xiamen, China, during 1987–2016. In the formula, y represents the value of AV (km), and x refers to the time (year).

As shown in Figure 3, during 1987–1997, the mean AV degraded rapidly, with a declining rate of −0.667 km/year. From 1997–2012, the AV decline was relatively steady, with a rate of −0.009 km/year. Since 2012, the AV in Xiamen showed a clear decreasing tendency, with rate of −0.612 km/year.

**Figure 4:**
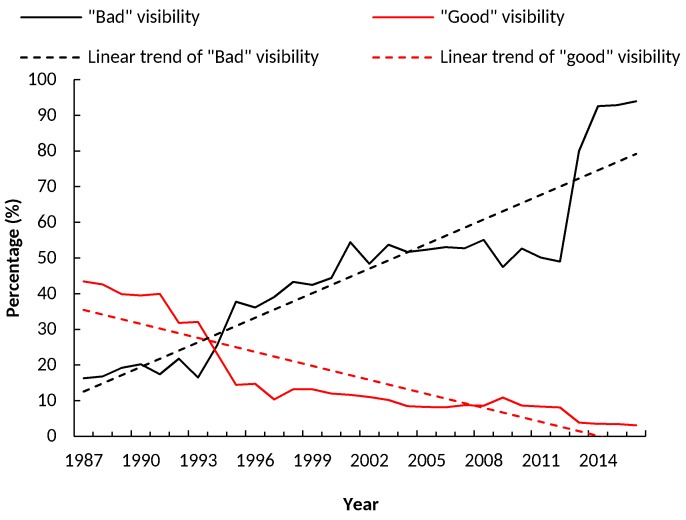
Annual percentages (%) of “bad” atmospheric visibility (<10 km) and “good” visibility (≥20 km) in Xiamen, during 1987–2016 (dashed lines refer to the curves of linear regression for the corresponding lines of trend).

In Figure 4, the annual percentages of the “bad” and “good” AV were calculated. During 1987–2016, the AV of Xiamen showed a decline in the percentage of “good” AV, with a declining rate of −1.31% per year, and revealed a rising trend in the percentage of “bad” AV, with a growth rate of 2.30% per year.

#### 3.1.2. Socioeconomic Factors

**Figure 5:**
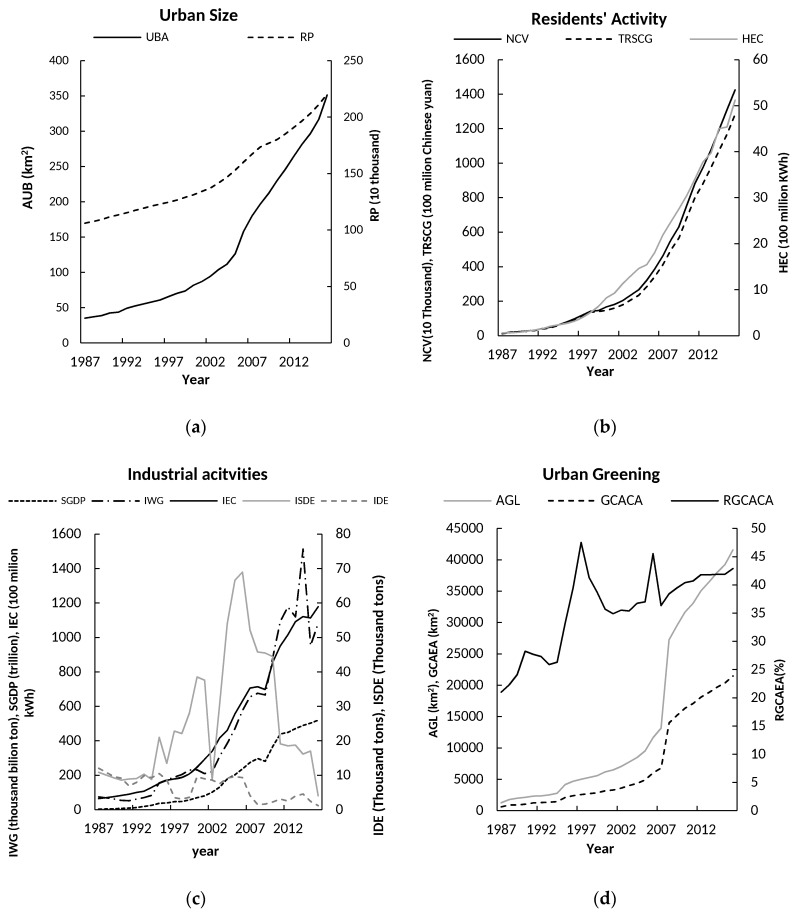
Variation of annual mean value of the socioeconomic factors of Xiamen, China during 1987–2016: (**a**) urban size (e.g., resident populations (RP), urban built-up areas (UBA)); (**b**) residents’ activities (e.g., total retail sales of consumer goods (TRSCG), number of civilian vehicles (NCV), household electricity consumption (HEC)); (**c**) industry activities (e.g., secondary industry gross domestic product (SGDP), industrial waste gas (IWG), Sulphur dioxide emissions (SDE), industrial dust emissions (IDE), industrial electricity consumption (IEC)); and (**d**) urban greening (e.g., green covered area of entire area (GCAEA), rate of green covered area of entire area (RGCAEA), area of green land (AGL)).

Overall, the indicators of urban size, residents’ activities, industrial activities, and urban greening showed growth trends (Figure 5). However, the Sulphur dioxide emissions (SDE), industrial dust emissions (IDE), and the rate of green covered area as part of the overall urban area (RGC) showed different trends. The SDE and IDE displayed obvious fluctuations (i.e., the first peak occurred in 2002, then dropped to a low value in 2003, then showed rapid growth between 2004 to 2006, and declined after 2007). The RGC showed an increasing trend, but there were obvious fluctuations, with three peaks in 1990, 1997, and 2006. The subsequent changes in the RGC could be related to the expansion of urban built-up areas and damage caused by typhoons.

### 3.2. Model Analysis

#### 3.2.1. Model Validation

The Pearson correlation analysis was conducted to examine the correlations between the socioeconomic factors and AV. All thirteen indicators had significant correlations with the indicators of the AV (Table 3). Nevertheless, indicators of urban greening (UG) (e.g., green covered area of entire area (GCACA), rate of green covered area of entire area (RGCACA), and area of green land (AGL)) were found to have negative relationships with the indicators of AV. Previous studies revealed UG as having positive effect on air pollution reduction. But in this study, the UG in Xiamen had limited effect on AV decline. Industrial dust emissions (IDE) and Sulphur dioxide emissions (SDE) had a positive correlation with the indicators of AV. This is contrary to common sense as well as findings from other studies [20,26,37,49,50]. It also means that the IDE and SDE in Xiamen did not significantly affect the AV, so we decided to remove these five indicators (GCACA, RGCACA, AGL, IDE, and SDE) from the model.

SPSS 23.0 was used to test the reliability of the exploratory factor analysis for the remaining eight socioeconomic indicators and the three AV indicators (Table 4). The results show that the Kaiser–Meyer–Olkin (KMO) value was 0.850, and the Bartlett sphere test approximated chi-square value was 909.423, both of which were higher than the lowest standard requirement of 0.6. The *p* value was 0.000 < 0.05, which reached a significant level, making the selected sample suitable for factor analysis.

According to the reliability test, the Cronbach’s coefficient (Cronbach’s α) of the four dimensions in this study ranged from 0.582 to 0.769, which was close to the standard value of 0.6, and indicated that the results had a good internal consistency. The minimum composite reliability (CR) value was 0.9032, which was higher than the lowest standard requirement of 0.6, and indicated that the observed variables had a good heterogeneity. The average variance extracted (AVE) values were from 0.7575 to 0.9801, which were higher than the standard threshold of 0.5, indicating that the observed variable can measure its underlying variables.

We used the method that was previously used in other studies [26] in order to modify the SEM (i.e., remove the indicators to find the best model). The urban size only had two observed indicators and was excluded from consideration. We considered the following nine conditions: resident secondary industry GDP removed (model B), industrial electricity consumption removed (model C), industrial waste gas removed (model D), total retail sales of consumer goods removed (model E), numbers of civilian vehicles removed (model F), household electricity consumption (model G), annual mean value of AV removed (model H), good AV rate removed (model I), and bad AV rate removed (model J). The chi-square (*χ*^2^) for the model was also called the likelihood ratio chi-square, or the chi-square goodness of fit. If the chi-square was relatively small, the model was regarded as acceptable when the value of *χ*^2^ divided by the degrees of freedom (df) was smaller, which means that the model is the better. The model had lower values for *χ*^2^, *χ*^2^/df, the Akaike information criterion (AIC), and the Browne–Cudeck criterion (BCC), and higher values for the normed fit index (NFI), incremental fit index (IFI), and comparative fit index (CFI); a higher R^2^ (squared multiple correlations) meant that the model was the better model. The values of the fit indicators showed that model D had a better fit (Table 5). Model D had the least *χ*^2^, *χ*^2^/df, AIC, and BCC, and the highest NFI, IFI, and CFI, as well as a relatively high R^2^ among the nine models. This meant that model D was the best model out of the nine models. It should be clear that with an increased data accuracy and longer period of study, the model selection could change.

**Figure 6:**
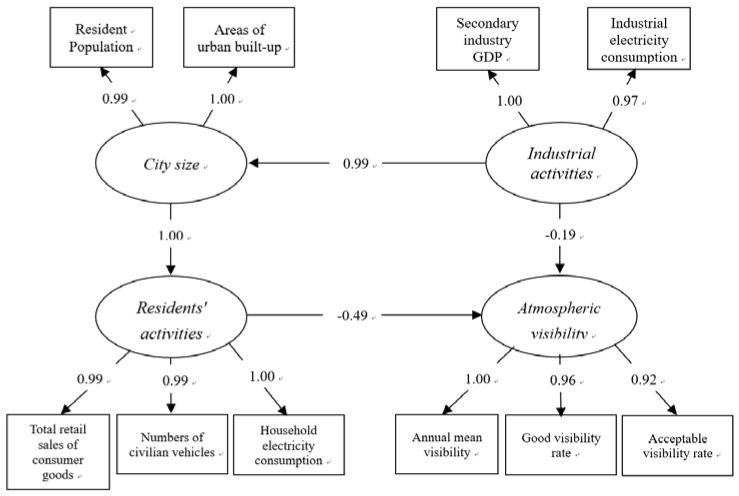
Standardized estimates of the modification model. The variables in the rectangles are the observed factors; the variables in the ellipses are the latent variables; the numbers on the arrows of the latent variables to the observed variables (latent variables) indicated the level of influence from the latent variable observed factors or the latent variables.

In the final model (Figure 6), the industrial waste gas was removed, because the model was found to have a better fit when the indicator was removed. The detailed fitting index is explained in Table 5.

#### 3.2.2. Modeling Results

The direct and indirect pathways through the urban sizes, industrial activities, and residents’ activities that effects the AV are shown in Figure 6. Overall, the three variables included in our model could explain the 46.2% of variation in AV (Figure 6). Industrial activities impact AV more than residents’ activities and urban size. From the direct impact point of view, the contribution of residents’ activities to the reduction of AV was higher than that of industrial activities; from the perspective of indirect effects, the role of urban size was higher than that of industrial development (Table 6).

Among all of the indicators, the number of resident vehicles (NRV), total retail sales of consumer goods (TRS), and the household electricity consumptions (HEC) were found to have the highest negative direct impact on AV (Table 7). The resident population (RP), urban built-up areas (UBA), secondary industry GDP (SGDP), and industrial electricity consumption (IEC) were the highest indirect impact factors.

## 4. Discussion

### 4.1. The Role of Residents’ Activities on Atmospheric Visibility in Xiamen

In Xiamen, residents’ activities contributed more as a direct impact on AV decline than urban size and industrial activities (Table 6). This was different from previous studies in other cities in China, because it found that industrial activities were the primary direct impact factors [19,34,51]. This may be because of Xiamen’s economic structure and the government’s strict restrictions, where the industry focuses on low pollution and low energy consumption (Xiamen Yearbook, 2005). The residents’ activities mainly included vehicle exhaust, household electricity consumption, and the total retail sales of consumer goods. Vehicle exhaust was referred as one of the three air pollution sources in urban areas. Kuo et al. (2013) found that great amounts of NOx were emitted from vehicles in urban areas, thus causing great amounts of nitrates to be formed in urban locations during stagnant weather. Their results highlighted the fact that vehicle exhaust plays a leading role in AV degradation in urban areas in central Taiwan [18]. In Xiamen city, the population and vehicle densities are relatively high; therefore, vehicle exhaust gas is one of the primary pollutants. Relevant studies have found that air pollutants reach peak values during the morning and evening peaks, when traffic is busy, which is consistent with the results of this study [52].

Household electricity consumption was used to reflect the density of the urban population and the intensity of the residents’ activities. Previous studies in China also concluded that the areas that consumed more electricity displayed heavier air pollutants [16,28,29]. Mo et al. (2013) revealed that thermal power is still the main source of electricity in China. Thermal power accounted for 75.56% and 73.44% of the sources of electricity in 2005 and 2010, respectively. In addition, the firepower electricity industry discharged 36.1% of the total fine particulate matter emissions in 2010, which indicated that the electric consumption was heavily correlated with air pollutant emissions [53].

The indicator of the total retail sales of consumer goods was related to the residents’ lives. According to the official data released in 2015, the top three items of total household consumption were food (29.7%), housing energy consumption (21.6%), and transportation (13.5%). Previous studies revealed that cooking smoke, housing energy consumption, and vehicle emissions are the main source of air pollutants in urban areas [32,54], which confirms our result listed in Section 3.2.2.

### 4.2. The Role of Industrial Activities on Atmosphercic Visibility in Xiamen

From the results of the model construction, industrial activities were identified as having the greatest impact on AV, but they were not the primary direct factor. The indirect impact of industrial activities on AV is −0.516, which had a direct effect of −0.159. Industrial activity has brought economic growth and thus has attracted populations, which effected urban expansion and residents’ activity (Table 7). Therefore, the indirect contribution of industrial pollution was relatively high.

The Xiamen City Environmental Protection Regulations (XMEPR) have been implemented since 2004, and these have led to a downward trend in the emission of SO_2_ and particulate matters (PMs) (Xiamen Statistical Yearbook 2005). Previous studies showed that SO_2_ and PMs are the most important factors affecting atmospheric AV [8,18,20,34,37]. With the reduction SO_2_ and PM emissions, industrial exhaust emissions are no longer the main factors that affect atmospheric AV in Xiamen [55,56,57]. This showed that the control of industrial gas emissions could have high effects on environmental quality improvement [55,56,57].

SGDP can reflect industrial output, industrial waste, and employment. For this reason, the industrial output value has an impact on AV. The industrial output value can directly reflect the intensity of industrial production in local cities. In certain regions, the impact of industrial intensity, that exceeds a certain limit, on air pollution will increase significantly.

Our results also revealed that industrial activities had indirect influences on declining AV, by influencing the city size and residents’ activities (Figure 3). The increase of industrial activities can lead to the expansion of cities through the construction of new factories and housing for workers. The increased industrial output can increase the availability and affordability of goods, which in turn can increase consumption by urban residents.

### 4.3. The Role of Urban Size on Atmospheric Visibility in Xiamen

City size also had significant effect on the AV decline in Xiamen. Our results indicated that the influence of city sizes on AV decline was mainly through the influence of residents’ activities (Figure 6). Large city sizes corresponded to a higher population and large urban built-up areas. Large urban built-up areas were associated with more construction activities, more traffic, and more energy consumption. All of these activities contributed to the decrease of AV [19,21,34]. These are the reasons for the urban built-up areas being considered as the “source” cause of AV decline. The significant influence of the resident population may be due to the fact that extra populations led to a greater energy consumption, as mentioned in Section 4.1. More people can also lead to more activities that can emit air pollutants, as discussed in Section 3.1. It has been observed that low AV in China often occurred in cities with large populations, such as Being, Shanghai, Chengdu, and Shenyang [8,19,25,34]. Our findings were in agreement with previous observations.

### 4.4. The Role of Urban Greening on Visibility in Xiamen

We found that there was a negative correlation between the index of UG and AV change, which was contrary to previous studies [22,23,24,44]. This phenomenon may relate to rapid urban expansion, which was found to be beyond the reduction pollution ability of UG [22,23,24,44], and the uneven distribution of UG and the air pollution level. Irga et al. (2015) concluded that urban areas with proportionally higher concentrations of UG may experience better air quality with regards to reduced ambient particulate matter; however, conclusions about other air pollutants have yet to be elucidated [49]. Pelkonen et al. (2017) suggested that in terms of the anthropogenic pollutants measured in northern climates, the role of UG is negligible in improving local air quality [50]. Grundström et al. (2014) even indicated that the effect of UG on air pollution concentrations was small [54].

From 1987 to 2016, the urban area of Xiamen showed a trend of expansion. The ever-expanding urban areas have caused the erosion of farmland, forest land, and grassland in the surrounding areas. As a result, the overall green space (including the urban green space, farmland, and forest land) declined. Chao et al. (2015) analyzed the characteristics of the land use types in Xiamen, including the urban areas and rural areas, and concluded that the “green land” (including urban green space, farmland, and forest land) showed a declining trend, a finding that supports our conclusion. They even found that although the urban green space in Xiamen had continued to expand, the urban green space of the core urban areas, where the AV monitoring sites are located, actually showed a slight downward trend [53]. This can be another contributing factor for the continuous decline in AV.

### 4.5. The Joint Contribution of Socioeconomic Factors

The socioeconomic factors included in this study contributed to a 46.2% variation in AV, indicating that these socioeconomic factors can better explain the changes in AV. In addition to the socioeconomic factors, meteorological factors can have an impact on AV. Previous work has revealed that low AV is associated with low wind speed, high humidity, and low temperature [8,19,37]. Other cities adjacent to Xiamen that produce pollutants can also affect the AV in Xiamen. In the early stages of the agricultural market in Zhangzhou, straw was often burnt. Studies showed that the burning of straw was one of the main factors that affected the AV of the atmosphere [19,20,21]. During the development of its industry, Quanzhou, the city with the highest GDP in the province of Fujian, had a heavy impact on the reduction of atmospheric AV. This impact even affected the AV in Xiamen.

### 4.6. Policy Implications

Xiamen has made many environment policies aimed at reducing air pollution and AV. For instance, the municipal government stipulated that the “Environmental Protection Regulations of Xiamen City” of 3 June 2004 should implement a sewage permit system for industrial enterprises at all levels, increase financial input for pollution control, control emission targets, and pay pollutant discharge fees to polluters, according to law. Overall, the effect of these environment policies has been positive, and it has contributed to the control of industrial pollution’s impact on the reduction of AV.

We obtained some results on the changes in the AV characteristics of Xiamen in the past 30 years, as well as the impact of comprehensive socioeconomic factors on AV. It has been shown that the residents’ activities in Xiamen have had the greatest direct impact on the reduction of AV, and that industrial pollution has also had a direct impact on the reduction of AV. Industrial activities and urbanization were found to have major indirect effects on the reduction of AV. According to our result, the main sources of direct pollution of vehicle exhausts, household electricity consumption, and the residents’ living exhausts were not reflected in the relevant regulations. Policy makers should focus on reducing the air pollution generated by residents’ activities, with the aim of increasing AV. Specific actions to achieve this could include promoting the purchase of environmentally friendly vehicles, natural gas vehicles, electric vehicles, and hybrid vehicles, in order to reduce the pollution caused by automobile exhaust gas; advocating for the usage of photovoltaic power generation, tidal power generation, nuclear power, and so on, reducing the air pollution brought about by coal-fired power generation; increasing the supply of energy-efficient natural gas; and advocating for the use of pollution-free energy sources in order to reduce the impact of the residents’ lives on air pollution.

### 4.7. Limitations

In this study, we revised the mature SEM model constructed by previous studies, then analyzed the relationship between the AV and socioeconomic factors in Xiamen during 1987−2016, as well as providing support for the Xiamen policy makers.

However, this study has several limitations. Firstly, the SEM model required a large amount of statistical data for this analysis. Because of the limitation of the government’s statistical data, the socioeconomic data of Xiamen City could only be used from the past 30 years. Secondly, the statistical data used by the model construction, such as the green space statistics and so on, came from the Chinese government and cannot be verified. Finally, we only collected and analyzed the data based on the annual data, which inevitably led to omissions. This part of the study remains to be data mined and further analyzed. In the future, related studies need to add more parameters to the SEM model so as to enhance our understanding of the mechanisms at work, such as other socioeconomic factors (including, e.g., residents’ education level) and the relationship between wind speed, humidity, temperature and other climatic factors, waste classification processing, and the residents’ education level. This will not only increase the richness of the data, but will also lead to more practical results.

## 5. Conclusions

An analysis of the relationship between the AV and socioeconomic factors in a longer-term perspective can lead to a comprehensive understanding of air pollution. Our results showed that the mean value of AV in Xiamen was 12.00 km, with a declining rate of −0.315 km/year during 1987−2016. The socioeconomic indicators of urban size, residents’ activities, industrial activities, and urban greening showed growth trends. However, SDE, IDE, and RGCAEA showed no significant trends during the study period. Based on the SEM model, we analyzed the trends and characteristics of AV and the socioeconomic factors in Xiamen during 1987–2016. We found that industrial activities contributed more to the decline in AV, followed by residents’ activities and urban size. In addition, the residents’ activities were the most significant factor directly affecting the AV. For all of the indicators, the residents’ electricity consumption and residents’ vehicle exhaust gases were the most influential direct influence factors. Based on the analysis results, this article evaluated the environmental protection policy of Xiamen City and provided suggestions and data support for the formulation of environment policies for a later period. This study partially revised the mature model of the predecessor [26] and made the results of the model significant and more reliable.

## Figures and Tables

**Table 1:** Selected indicators and related sources.

Indicator	Effect	Sources
City size		
Urban built-up areas (UBA)	Negative	[33,34]
Resident populations (RP)	Negative	[33,35,36]
Industrial activities		
Secondary industry gross domestic product (SGDP)	Negative	[8,20,33]
Industrial waste gas (IWG)	Negative	[8,19,20,21,37]
Industrial dust emissions (IDE)	Negative	[25,34,38]
Sulphur dioxide emissions (SDE)	Negative	[25,34,38]
Industrial electricity consumption (IEC)	Negative	[39,40]
Residents’ activities		
Numbers of civilian vehicles (NCV)	Negative	[18,19,20,33,41]
Total retail sales of consumer goods (TRSCG)	Negative	[19,32,42]
Household electricity consumption (HEC)	Negative	[32,43]
Urban greening		
Green covered area of completed area (GCACA)	Positive	[23,44,45,46]
Rate of green covered area of completed area (RGCACA)	Positive	[23,44,45,46]
Area of green land (AGL)	Positive	[22,24,46,47,48]

**Table 2:** Mean annual atmospheric visibility (km) of six periods and the change trends (km/year) of Xiamen, China.

	1987–1991	1992–1996	1997–2001	2002–2006	2007–2011	2012–2016	1987–2016
Mean Visibility	17.14	13.92	11.52	10.54	10.55	8.33	12.00
Standard Deviation	±0.3395	±1.6692	±0.3061	±0.3603	±0.3811	±1.2518	±2.9909
Trend	−0.15	−0.98	0.02	−0.20	0.001	−0.61	−0.315

**Table 3:** Pearson correlation of indicators selected in the model.

	**Resident Populations** **(RP)**	**Urban Built-Up Areas** **(UBA)**	**Industrial Electricity Consumption** **(IEC)**	**Secondary Industry GDP** **(SGDP)**	**Industrial Waste Gas** **(IWG)**	**Industrial Dust Emission** **(IDE)**	**Sulphur Dioxide Emission** **(SDE)**
Annual mean AV	−0.846 **	−0.798 **	−0.821 **	−0.797 **	−0.778 **	0.625 **	0.410 *
Good AV rate	−0.762 **	−0.702 **	−0.733 **	−0.706 **	−0.688 **	0.596 **	0.486 **
Bad AV rate	−0.587 **	−0.582 **	−0.600 **	−0.586 **	−0.636 **	0.447 *	0.223
	**Total Retail Sales of Consumer Goods** **(TRSCG)**	**Numbers of Civilian Vehicles** **(NCV)**	**Household Electricity Consumption** **(HEC)**	**Green Covered Area of Completed Area** **GCACA**	**Rate of Green Covered Area of Completed Area** **(RGCACA)**	**Area of Green Land** **(AGL)**	
Annual mean AV	−0.768 **	−0.770 **	−0.803 **	−0.740 **	−0.880 **	−0.740 **	
Good AV rate	−0.658 **	−0.654 **	−0.703 **	−0.647 **	−0.896 **	−0.647 **	
Bad AV rate	−0.563 **	−0.556 **	−0.582 **	−0.554 **	−0.509 **	−0.552 **	

* means the *p* value < 0.05, ** means the *p* value < 0.01.

**Table 4:** Test of measurement scale. CR—composite reliability; AVE—average variance extracted.

Latent Variable	Measurement Items	Factor Loadings	AVE	CR	Cronbach’s α
Urban size	Resident population (RP)	0.994	0.9801	0.99	0.769
	Urban built-up areas (UBA)	0.986			
Industry	Secondary industry GDP (SGDP)	0.984	0.9553	0.9846	0.603
	Industrial waste gas (IWG)	0.961			
	Industrial electricity consumption (IEC)	0.987			
Resident’s activities	Total retail sales of consumer goods (TRSCG)	0.974	0.9578	0.9855	0.582
	Numbers of civilian vehicles (NCV)	0.974			
	Household electricity consumption (HEC)	0.988			
Visibility	Annual mean visibility (AMV)	−0.881	0.7575	0.9032	0.759
	Good visibility rate (GVR)	−0.797			
	Bad visibility rate (BVR)	−0.928			

**Table 5:** Comparison of fitting results among models. AIC—Akaike information criterion; BCC—Browne–Cudeck criterion; NFI—normed fit index; IFI—incremental fit index; CFI—comparative fit index.

Fitting Indicators	*χ* ^2^	*χ*^2^/df	AIC	BCC	NFI	IFI	CFI	R^2^
Model A	-	-	-	-	-	-	-	-
Model B	-	-	-	-	-	-	-	-
Model C	173.555	5.599	241.555	283.111	0.837	0.862	0.859	0.450
Model D	154.103	4.971	222.103	254.625	0.852	0.878	0.875	0.462
Model E	-	-	-	-	-	-	-	-
Model F	158.181	5.103	226.181	267.736	0.852	0.878	0.875	0.453
Model G	-	-	-	-	-	-	-	-
Model H	-	-	-	-	-	-	-	-
Model I	-	-	-	-	-	-	-	-
Model J	-	-	-	-	-	-	-	-

Note: “-” represents this result is not significant, R^2^ means how much the model can explain the variation in visibility.

**Table 6:** The influence of socioeconomic variables on visibility in Xiamen, China 1987–2016.

Socioeconomic Variables	Normalized Coefficient		
	Direct Influence	Indirect Influence	Total Influence
Industrial activities	−0.159	−0.516	−0.675
Urban size	0	−0.522	−0.522
Residents’ activities	−0.523	0	−0.523

**Table 7:** Effects of socioeconomic indicators on atmospheric visibility in Xiamen during 1987–2016.

Socioeconomic Factors	Indicators	Normalized Coefficient		
		Direct	Indirect	Total
Urban size	Resident populations	0	−0.244	−0.244
	Urban built-up areas	0	−0.246	−0.246
Industrial activities	Secondary industry GDP	−0.096	−0.246	−0.342
	Industrial electricity consumption	−0.093	−0.239	−0.332
Residents’ activities	Total retail sales of consumer goods	−0.163	0	−0.163
	Household electricity consumption	−0.164	0	−0.164
	Numbers of civilian vehicles	−0.163	0	−0.163
